# Activity Tracker–Based Metrics as Digital Markers of Cardiometabolic Health in Working Adults: Cross-Sectional Study

**DOI:** 10.2196/16409

**Published:** 2020-01-31

**Authors:** Yuri Rykov, Thuan-Quoc Thach, Gerard Dunleavy, Adam Charles Roberts, George Christopoulos, Chee-Kiong Soh, Josip Car

**Affiliations:** 1 Centre for Population Health Sciences Lee Kong Chian School of Medicine Nanyang Technological University Singapore Singapore; 2 School of Mechanical and Aerospace Engineering College of Engineering Nanyang Technological University Singapore Singapore; 3 Division of Leadership, Management and Organisation Nanyang Business School, College of Business Nanyang Technological University Singapore Singapore; 4 School of Civil and Environmental Engineering College of Engineering Nanyang Technological University Singapore Singapore

**Keywords:** mobile health, metabolic cardiovascular syndrome, fitness trackers, wearable electronic devices, Fitbit, steps, heart rate, physical activity, circadian rhythms, sedentary behavior

## Abstract

**Background:**

Greater adoption of wearable devices with multiple sensors may enhance personalized health monitoring, facilitate early detection of some diseases, and further scale up population health screening. However, few studies have explored the utility of data from wearable fitness trackers in cardiovascular and metabolic disease risk prediction.

**Objective:**

This study aimed to investigate the associations between a range of activity metrics derived from a wearable consumer-grade fitness tracker and major modifiable biomarkers of cardiometabolic disease in a working-age population.

**Methods:**

This was a cross-sectional study of 83 working adults. Participants wore Fitbit Charge 2 for 21 consecutive days and went through a health assessment, including fasting blood tests. The following clinical biomarkers were collected: BMI, waist circumference, waist-to-hip ratio, blood pressure, triglycerides (TGs), high-density lipoprotein (HDL) and low-density lipoprotein cholesterol, and blood glucose. We used a range of wearable-derived metrics based on steps, heart rate (HR), and energy expenditure, including measures of stability of circadian activity rhythms, sedentary time, and time spent at various intensities of physical activity. Spearman rank correlation was used for preliminary analysis. Multiple linear regression adjusted for potential confounders was used to determine the extent to which each metric of activity was associated with continuous clinical biomarkers. In addition, pairwise multiple regression was used to investigate the significance and mutual dependence of activity metrics when two or more of them had significant association with the same outcome from the previous step of the analysis.

**Results:**

The participants were predominantly middle aged (mean age 44.3 years, SD 12), Chinese (62/83, 75%), and male (64/83, 77%). Blood biomarkers of cardiometabolic disease (HDL cholesterol and TGs) were significantly associated with steps-based activity metrics independent of age, gender, ethnicity, education, and shift work, whereas body composition biomarkers (BMI, waist circumference, and waist-to-hip ratio) were significantly associated with energy expenditure–based and HR-based metrics when adjusted for the same confounders. Steps-based interdaily stability of circadian activity rhythm was strongly associated with HDL (beta=5.4 per 10% change; 95% CI 1.8 to 9.0; *P*=.005) and TG (beta=−27.7 per 10% change; 95% CI −48.4 to −7.0; *P*=.01). Average daily steps were negatively associated with TG (beta=−6.8 per 1000 steps; 95% CI −13.0 to −0.6; *P*=.04). The difference between average HR and resting HR was significantly associated with BMI (beta=−.5; 95% CI −1.0 to −0.1; *P*=.01) and waist circumference (beta=−1.3; 95% CI −2.4 to −0.2; *P*=.03).

**Conclusions:**

Wearable consumer-grade fitness trackers can provide acceptably accurate and meaningful information, which might be used in the risk prediction of cardiometabolic disease. Our results showed the beneficial effects of stable daily patterns of locomotor activity for cardiometabolic health. Study findings should be further replicated with larger population studies.

## Introduction

### Background

Wearable consumer-grade fitness trackers are becoming more widespread every year. The market and number of wearables are expected to more than double from an estimated 527 million devices worldwide in 2017 to more than 1.1 billion in 2022 [1], achieving a market size of US $27 billion by 2022 [2]. These wearables are equipped with multiple sensors and can monitor and record biometric and locomotor activity data, including steps, heart rate (HR), blood volume pulse, electrodermal activity, skin and body temperature, respiration rate, oxygen saturation, electrocardiography, and sleep patterns. Apart from providing direct information about an individual’s physical health status (eg, body temperature), some of these physiological and behavioral characteristics can be considered as risk factors or markers related to different diseases. For example, increased resting HR (RHR) is an important risk marker of cardiovascular disease [3,4], and insufficient physical activity (PA) is a risk factor of major noncommunicable diseases [5,6]. At the same time, activity metrics have potential value in risk prediction of other health conditions, including mental disorders [7,8] and neuropsychiatric illness [9]. As greater adoption of wearables can enhance personalized health monitoring, scale up population health screening, and facilitate early detection of some diseases, research should explore associations between metrics derived from consumer-grade wearables and clinical and biological health markers. Multisensor and continuous data available from fitness trackers at second-by-second or minute-by-minute resolution allow the retrieval of various metrics and exploration of their clinical significance. In this work, we focused on the association between wearable data and biomarkers of cardiovascular and metabolic diseases, which are the leading causes of mortality and disability worldwide [10].

### Related Work

The risk of sedentary behavior and the beneficial effects of PA for cardiometabolic disease have been extensively studied in different populations [11-22]. Large cross-sectional studies demonstrated reliable evidence that sedentary time and PA measured objectively with wearable accelerometers are strongly related to most cardiometabolic biomarkers, including waist circumference, BMI, high-density lipoprotein (HDL) cholesterol, triglycerides (TGs), fasting blood glucose (BG) level, high-sensitivity C-reactive protein (CRP), and blood pressure independently from major confounders. Most studies measured average daily duration of sedentary behavior and PA in minutes with research-grade actigraphs; however, the observation period in these studies did not exceed 7 consecutive days. Key differences in findings concern the significance of light-intensity PA and moderate-to-vigorous PA (MVPA) as protective factors for cardiometabolic disease independent of sedentary time. Different levels of PA were found to vary in significance in specific population groups. For example, light-intensity PA was found to be beneficial for cardiometabolic health in American Hispanic adults with type 2 diabetes [17], although it did not appear to have a beneficial effect in older adults without known diabetes [22]. However, the overall beneficial role of PA has not been questioned.

Several studies explored the association between rest/activity rhythms measured by wearables and indicators of cardiometabolic disease [23-28]. Continuous activity tracking permits the assessment of circadian patterns, revealing possible irregularities and disruptions. To our knowledge, there are only a few studies that have investigated the associations of regularity in activity rhythms, measured as interdaily stability (IS), with cardiometabolic risk or related biomarkers. Paudel et al [23] studied rest/activity rhythms in 2968 community-dwelling older men and found that lower regularity in circadian activity was associated with an increased risk of peripheral vascular disease events (such as acute arterial occlusion, rupture***,*** or dissection) independent of age, race, smoking status, walking for exercise, history of cardiovascular events***,*** and even a number of cardiovascular risk factors***,*** including diabetes, blood pressure, total cholesterol, and HDL cholesterol. Sohail et al [24] studied actigraphic data from 1137 older adults and found that IS was related to several key components of the metabolic syndrome. In particular, higher IS was associated with lower blood pressure, higher HDL cholesterol, lower risk of being obese (defined with BMI), or having diabetes (according to medical history) independent of total daily PA and other confounders. Another study did not find significant associations between IS and blood pressure and cholesterol level (including HDL) in patients with diabetes [25]. One more study reported the negative relationship between IS and systolic blood pressure (SBP) and diastolic blood pressure (DBP) [26]. In addition, two studies tested the association of IS with BMI, which was found to be significant in one study [27] and nonsignificant in the other [28].

Most research on consumer-grade fitness trackers with multiple sensors are intervention studies focused on their value in promoting PA in different populations [29-31] or monitoring studies of patients with different health conditions [32,33]. Despite great enthusiasm for fitness trackers, the current evidence does not consistently indicate significant or long-term effects of using wearables for the promotion of greater PA and habitual behavior change [34-38]. However, fewer studies have explored the associations between multidimensional data from fitness trackers and clinical and biological markers. Li et al [39] analyzed data collected with Intel Basis smartwatches from 43 individuals over the course of 152 days on average. Initially, the authors demonstrated that elevated HR and skin temperature were strongly associated with elevated CRP, a biomarker of inflammatory response. To identify transitions between healthy and ill states, they used fraction of outlying values of HR and skin temperature, which were calculated with the peak detection method. Second, the authors found that both daytime HR (DayHR) and difference between DayHR and nighttime HR (NightHR) were positively correlated with steady-state plasma glucose level, indicating differences between insulin-sensitive and insulin-resistant individuals. Price et al [40] analyzed comprehensive health data of 108 individuals, including activity tracking with Fitbit wearables. However, along with modest compliance of using wearables (only 64% of the participants met the criterion of a minimum of 40 days of observation), authors did not find any significant correlations between average energy expenditure (calories) and biomarkers. Lim et al [41] analyzed data from 233 volunteers, combining activity tracker data of 3 complete days and multiple cardiovascular and metabolic disease clinical markers. They found that higher RHR was significantly associated with most clinical markers, including higher BMI, waist circumference, DBP and SBP, TG and BG levels, and lower HDL cholesterol, whereas a higher step count was significantly associated only with lower BMI, waist circumference, and TG.

### Objectives

Wider adoption of consumer-grade fitness trackers compared with research-grade wearables stimulates the exploration and testing of activity metrics, which might be used for risk prediction of cardiometabolic disease. Compared with previous research, we, first, focused on working-age population; second, harnessed activity tracking for a longer period to analyze regularity of circadian activity rhythms; and, third, used fasting blood samples to get cardiometabolic biomarkers. This allowed us to go beyond standard metrics available from consumer-grade fitness trackers. Thus, the objective of this study was to investigate the associations between different activity metrics retrievable from a consumer-grade fitness tracker and major modifiable biomarkers of cardiometabolic disease, relying on prolonged activity monitoring.

## Methods

### Study Design and Participants

The data used in this paper were obtained from a workplace cohort study conducted in Singapore by the Nanyang Technological University (NTU) [42]. In total, four organizations agreed to join the study: two from the transport industry, a cooling plant, and a university. Healthy volunteers from these organizations were invited to participate in the study via meetings, workplace posters, and emails (for more details on the recruitment process, refer to the study by Dunleavy et al [42]). The NTU institutional review board approved the study protocol and informed consent form (IRB application reference: 2015/2601).

### Measurements

#### Health Outcomes: Cardiometabolic Disease Risk Biomarkers

In accordance with the American Heart Association guidelines [43,44], cardiometabolic syndrome is determined by an abnormal condition of the following characteristics: waist circumference (or waist-to-hip ratio), blood pressure, TG, HDL cholesterol, and BG. Hence, we considered the following clinical biomarkers in this study: BMI, waist circumference, waist-to-hip ratio, SBP, DBP, fasting levels of total cholesterol, HDL and low-density lipoprotein (LDL) cholesterol, TG, and BG.

Standardized self-report questionnaires were used to collect sociodemographic and health behavior characteristics. Trained staff performed clinical measurements, such as height, weight, and waist and hip circumferences, according to a standard protocol using standardized tools [15]. Height was measured using a stadiometer (Seca 217) to the nearest 0.1 cm, and weight was measured in light clothing using a digital scale (Seca 874) to the nearest 0.1 kg. Waist and hip circumferences were measured using a stretch-resistance tape (Seca 201). Waist circumference was measured at the midpoint between the lower margin of the last palpable rib and the top of the iliac crest (hip bone). Hip circumference was measured at the maximum circumference over the buttocks. BMI was calculated as weight in kilograms divided by the square of height in meters. Waist-to-hip ratio was calculated as the ratio between waist and hip circumferences. Blood pressure, in accordance with the National Health and Nutrition Examination Survey protocol [45], was measured over the right arm using an appropriate cuff size with an automatic digital blood pressure monitor (Dinamap Pro100V2; Criticon). The average value of the 3 readings taken with 2-min intervals was used.

Venous blood samples were collected from participants in a fasting state (at least 8 hours) by trained phlebotomists. A maximum of approximately 11 mL of blood was drawn into 2 tubes—8 mL in plain and 3 mL in fluoride tubes. Blood samples were transported immediately, in cooler boxes (4°C), to an internationally accredited laboratory for analysis. Blood samples were processed using the hexokinase method for plasma glucose and enzymatic methods for serum lipids on a COBAS 6000 analyzer, using kits supplied by Roche Diagnostics. LDL cholesterol was estimated using the Friedewald equation for those with TGs ≤ 4.52 mmol/L [46], whereas for the rest, values estimated by the direct method were used. Serum 25-hydroxyvitamin D concentrations were measured using the chemiluminescence immunoassay method on a Cobas e 411 analyzer with kits supplied by Roche Diagnostics.

#### Explanatory Variables: Activity Tracker–Based Metrics

Fitbit Charge 2 devices (a consumer-grade fitness tracker) were used in the study to collect activity data. The accuracy of Fitbit devices in collecting activity data has been tested in multiple studies [47-51]. According to a systematic review [49], studies have indicated that Fitbit wearables were likely to provide comparatively accurate (ie, similar to research-grade monitors) measures of step counts and sleep duration in free-living conditions. However, Fitbit wearables less accurately measure energy expenditure, underestimating sedentary time and overestimating time spent in MVPA [49,51].

Participants wore Fitbit devices on their wrists for a period of 23 days. As the first and the last days of wearing the fitness tracker were partial days, the total observation period was 21 days (3 weeks) of continuous tracking. Participants were instructed to wear the Fitbit tracker all day and to remove only when taking a shower or to charge it. In addition, participants were instructed to open the Fitbit mobile app and allow it to synchronize with the tracker once every mealtime and to not change any settings in the Fitbit app for the period of the study. To check the completeness of the activity tracking, we counted the number of hours per day with recorded HR data. Participants with a minimum of 14 days with greater than or equal to 18 valid hours each (maximum of 6 missing hours per day) were included for further analysis.

The fitness tracker records the data to the participant’s own Fitbit account through the official Fitbit mobile app on the participant’s mobile phone. The data were saved on Fitbit’s own server, and participants gave consent for Fitbit to send their data to our data collection server, after which the data were automatically retrieved and saved by the server. Our data collection server was situated within NTU’s network on a password-protected computer located within the Culture Science Institute’s Bio-Cognitive Laboratory. The server maintained a list of registered Fitbit accounts and sent daily requests through the Web application program interface to retrieve data from Fitbit’s own server database. Fitbit Charge 2 algorithmically derives and records the following variables from raw sensor data: steps, distance, elevation, calories, HR, and a number of sleep characteristics. Steps, distance, elevation, and calorie data are available at an intraday level in minute-by-minute intervals. Intraday HR data are available at 5- or 10-second intervals.

The following wearable-derived activity metrics were considered in the study: daily average steps, daily average HR, RHR, DayHR, NightHR, delta of RHR (dRHR), circadian delta of HR (cdHR), IS of locomotor activity, interdaily variation (IV) of locomotor activity, daily average sedentary time, and daily minutes of light-, moderate-, and vigorous-intensity PA.

To determine RHR, DayHR, and NightHR from the fitness trackers, we followed the approach proposed by Lim et al [41]. RHR was calculated as the average HR of 15-min intervals with less than or equal to 100 steps. DayHR was obtained by averaging HR values between 2 pm and 4 pm, whereas NightHR sampled time points between 2 am and 4 am. dRHR is the difference between average HR and RHR. cdHR is the difference between DayHR and NightHR.

We followed the common conception of sedentary behavior and defined sedentary time as “any waking behavior characterized by an energy expenditure ≤1.5 metabolic equivalents (METs), while in a sitting, reclining, or lying posture” [52]. Hence, to determine sedentary time, we excluded all sleep intervals and calculated a daily mean of total minutes with less than or equal to 1.5 METs.

**Figure 1:**
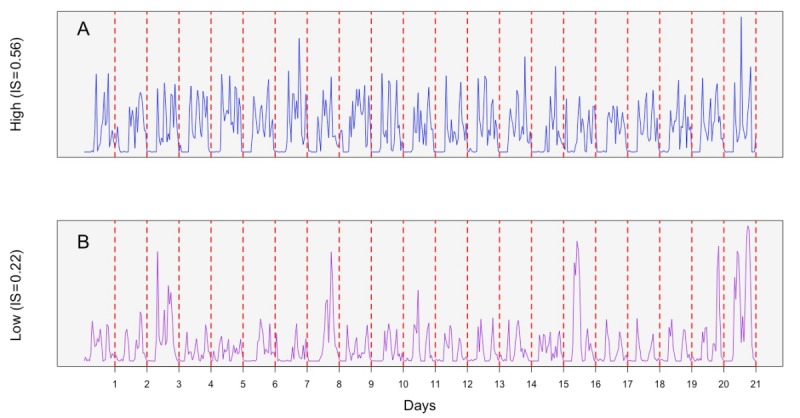
Steps tracking charts. Individuals with relatively high (A) and low (B) interdaily stability but similar total daily steps. x-axis: time in days. y-axis: steps count. IS: interdaily stability of locomotor activity rhythm.

IS is a nonparametric measure that evaluates the stability/similarity of activity patterns across a series of 24-hour cycles, that is, the extent to which an individual consistently follows some regular activity pattern from day to day. IS was calculated as the variance of the average steps-based 24-hour daily profile divided by the total variance of all days’ 24-hour profiles [53]. Higher IS indicates that an individual has a more stable circadian pattern over the course of observation period regardless of the overall level of activity, and lower IS indicates less stability in circadian activity distribution (see Figure 1).

To calculate IV of locomotor activity, we obtained a steps-based daily profile of coefficients of variation for each hour in a 24-hour cycle and took an average of these coefficients. Thus, IV indicates the average hour-by-hour variation of daily activity across the observation period regardless of the overall level of activity. We proposed this metric as alternative to IS, which aims to assess the same phenomena with a different approach.

The daily duration of light-intensity PA and MVPA was determined according to the PA guidelines of US Department of Health and Human Services [54], where moderate-intensity PA corresponds to energy expenditure from 3.0 to 6.0 METs, vigorous-intensity PA is above 6.0 METs, and light-intensity PA is below 3.0 METs. We sampled minutes within these intervals separately and took an average of the daily sum of these minutes.

### Data Analysis

We clustered participants according to their normalized average 24-hour profiles of steps using k-means cluster analysis (k=3) to identify groups of participants with similar activity patterns. Normalization of data allowed us to ignore individual differences in the absolute daily number of steps and focus on the temporal pattern of activity distribution. We used steps data for cluster analysis because step counts are probably the most accurate Fitbit metric [49].

An additional analysis was done to determine whether a longer period of tracking provides more stable estimates of RHR than estimates of RHR sampled from 3-day windows. For this analysis, we used the Kolmogorov-Smirnov test to compare the distribution of RHR values from a full period with the distribution of RHR values sampled from ten 3-day windows for each participant. Then the fraction of *failed* tests (with *P*<.05, meaning that distributions are significantly different) was calculated for each participant and on the study sample level.

Spearman rank correlation was used to explore preliminary associations between continuous activity metrics and continuous biomarker metrics. A correlation network based on Spearman coefficients [40] was plotted to display significant associations between activity metrics and clinical biomarkers.

Multiple linear regression analysis was used to determine the extent to which each metric of locomotor activity is associated with clinical biomarkers. For all models, adjustments were made for age, gender, ethnicity (3 categories: Chinese, Malay, or Indian), education, and shift work (shift worker or not). Thus, each regression model includes one explanatory variable (activity tracker–based metric) and aforementioned covariates. In addition, pairwise multiple linear regression analysis was used to investigate the significance and mutual dependence of activity metrics when two or more of them were found to be significant predictors of the same outcome from the previous step of the analysis. Pairwise multiple regression was used instead of including all predictors at once because the sample size limits the number of predictors that can be included in a regression model according to the *one in ten* rule of thumb (minimum 10 cases per predictor). Linear model (*lm*) function in R was used to execute computation (see Multimedia Appendix 1 for the R code used for data processing and analysis).

## Results

### Characteristics of Participants

**Figure 2:**
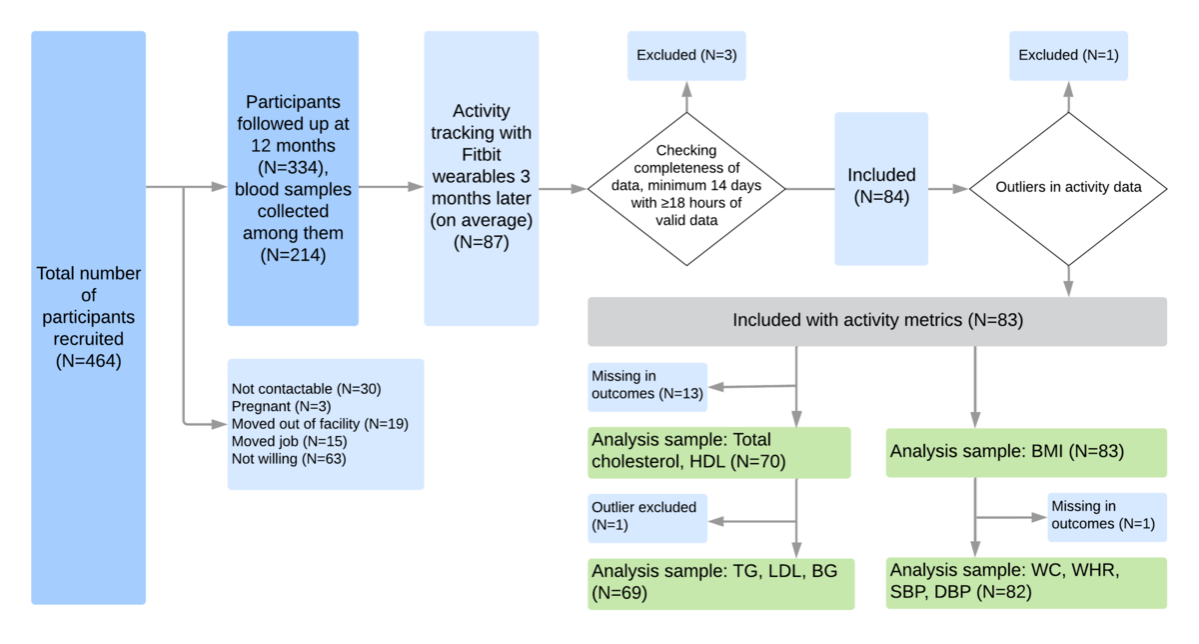
Participants flow diagram. BG: blood glucose; DBP: diastolic blood pressure; HDL: high-density lipoprotein; LDL: low-density lipoprotein; SBP: systolic blood pressure; TG: triglyceride; WC: waist circumference; WHR: waist-to-hip ratio.

A total of 464 full-time employees (aged ≥21 years) were recruited and enrolled in the study (Figure 2). Of these, 334 participants were followed up at 12 months, and blood samples were collected from 214 of them. Three months later, 87 volunteers were issued with consumer-grade fitness trackers to continuously record their biometric and locomotor activity data. These participants were randomly selected among those who confirmed they were going to be at work for 3 weeks (ie, not planning to be on vacation or on a business trip). In total, 87 participants were tracked for 21 days. Three participants were excluded because of incomplete activity data (see Table 1). One participant was excluded from the sample because of extremely high daily locomotor activity (average daily steps above 24,000) compared with others. Thus, we had 83 participants with activity data eligible for further analysis. In addition, there was 1 participant with missed waist circumference and blood pressure measurements. One outlier was excluded in the regression models of TG, BG, and LDL. The final sample for body composition measures was 83 (or 82 for some outcomes because of missing data), and 70 for blood test analysis (or 69 without the outlier).

Table 2 summarizes the characteristics of participants. The mean age of participants was 44.3 years (range 22-65 years), and the majority of them were male (64/83, 77%). The average step count was 10,865 steps per day (median 10479.4), and HR and RHR were 76 (median 75) and 70 (median 69), respectively (see Multimedia Appendix 2 for the differences in activity-tracker-based metrics between shift and nonshift workers).

**Table 1:** The distribution of the number of valid days (1702 days in total) among participants (N=84).

Number of valid days	Number of participants
21	53
20	15
19	10
18	3
16	1
15	1
14	1

**Table 2:** Summary statistics of participants.

Variable			Values	
**Sociodemographic characteristics**				
	Age (years; n=83), mean (SD)			44.3 (11.9)
	**Gender, n (%)**			
		Male		64 (77)
		Female		19 (23)
	**Ethnicity, n (%)**			
		Chinese		62 (75)
		Indian		13 (16)
		Malay		8 (9)
	**Education, n (%)**			
		Below university degree		54 (65)
		University degree		29 (35)
	**Shift worker, n (%)**			
		No		42 (51)
		Yes		41 (49)
**Health outcomes: cardiometabolic disease biomarkers, mean (SD)**				
	BMI (kg/m^2^; n=83)			24.6 (4.4)
	Waist circumference (cm; n=82)			82.9 (13.2)
	Waist-to-hip ratio (n=82)			0.86 (0.08)
	Systolic blood pressure (mm Hg; n=82)			119.9 (13.6)
	Diastolic blood pressure (mm Hg; n=82)			71.8 (10.7)
	Total cholesterol (mg/dL; n=70)			215.7 (34.6)
	High-density lipoprotein (mg/dL; n=70)			57.5 (15.7)
	Low-density lipoprotein (mg/dL; n=69)			129.1 (27.5)
	Triglyceride (mg/dL; n=69)			133.4 (77.4)
	Blood glucose (mg/dL; n=69)			96.6 (14.6)
**Explanatory variables: activity tracker–based metrics (n=83), mean (SD)**				
	Steps (per day)			10,865 (2775)
	Interdaily stability of locomotor activity rhythm			0.28 (0.11)
	Interdaily variation of locomotor activity			1.31 (0.23)
	Heart rate (bpm)			76.3 (7.6)
	Resting heart rate (bpm)			70.6 (8.1)
	Delta of resting heart rate (bpm)			5.7 (1.9)
	Daytime heart rate (bpm)			81.5 (9.1)
	Nighttime heart rate (bpm)			64.1 (8.1)
	Circadian delta of heart rate (bpm)			17.4 (5.7)
	Sedentary time (min/d)			787.9 (99.3)
	Light-intensity PA^a^ (min/d)			1202.9 (60.2)
	Moderate-intensity PA (min/d)			205.4 (55.1)
	Vigorous-intensity PA (min/d)			31.6 (16.1)
	Moderate-to-vigorous PA (min/d)			237.1 (60.2)

^a^PA: physical activity.

**Figure 3:**
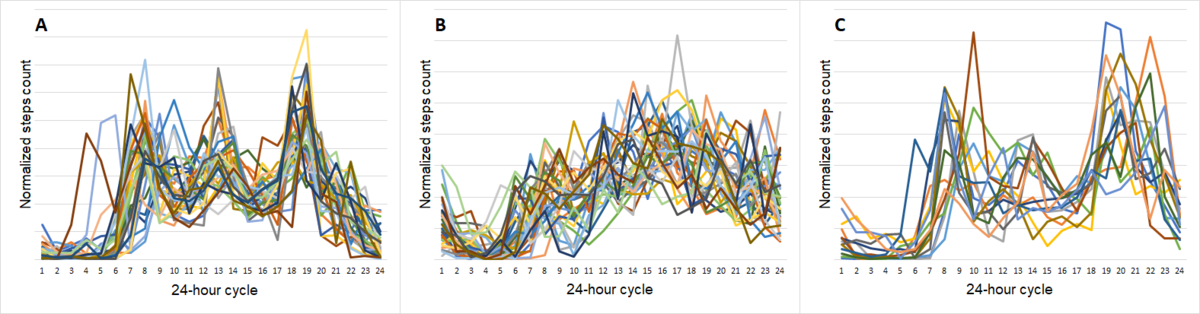
Visualization of normalized average 24-hour activity profiles by clusters. A: first cluster (N=35); B: second cluster (N=34); C: third cluster (N=14). Each line represents an individual within a cluster. x-axis: hours in a daily cycle. y-axis: average number of steps normalized by an individual sum of steps in average 24-hour profile.

We obtained 3 groups of sizes—35, 34, and 14 individuals, respectively—using k-means cluster analysis. Visualization of normalized 24-hour activity profiles (Figure 3) showed that clusters differ in the number of activity peaks, having 3 (morning, lunch, and evening), 2 (morning and evening), or 0 clear peaks throughout a day. The absence of clear peaks in the average activity profiles from the B cluster might be the result of both stable and evenly distributed activity or irregularity and inconsistency in the daily activity of the participants from this cluster. As data clustering indicated a meaningful division of participants, cluster membership was also used as a single categorical predictor of cardiometabolic disease biomarkers in further analysis.

Finally, an additional analysis of stability of RHR indicated that RHR estimated within 3-day windows is different from RHR estimated from the full period (3 weeks) in half of the cases. The sample level fraction of *failed* Kolmogorov-Smirnov tests was 0.49 (*P* values of all Kolmogorov-Smirnov tests are presented in Multimedia Appendix 3). Therefore, we used RHR estimated from the full period in further analysis.

### Associations Between Activity Tracker Metrics and Cardiometabolic Disease Biomarkers

**Figure 4:**
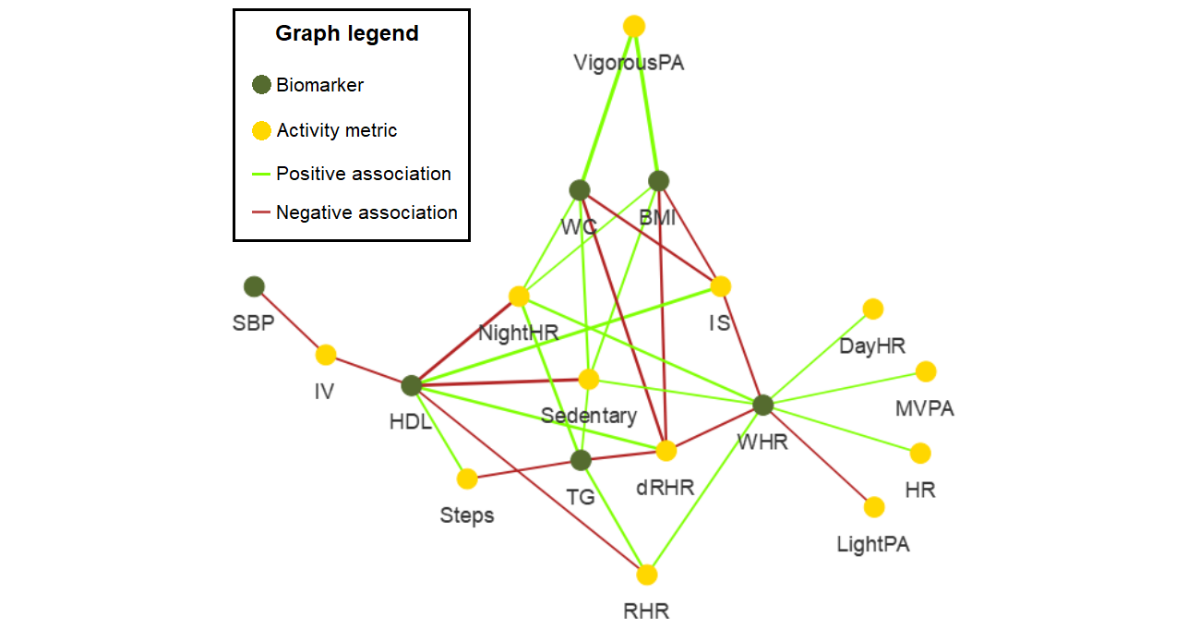
Correlation network of cardiometabolic disease biomarkers and activity tracker metrics. Only associations significant at *P*<.05 were displayed. Width of lines is proportional to absolute value of Spearman coefficients that vary between 0.22 and 0.38. BG: blood glucose; DayHR: daytime heart rate; DBP: diastolic blood pressure; dRHR: delta of resting heart rate; HDL: high-density lipoprotein; HR: heart rate; IS: interdaily stability of locomotor activity rhythm; IV: interdaily variation of locomotor activity; LDL: low-density lipoprotein; MVPA: moderate-to-vigorous physical activity; NightHR: nighttime heart rate; PA: physical Activity; RHR: resting heart rate; SBP: systolic blood pressure; TG: triglyceride; WC: waist circumference; WHR: waist-to-hip ratio.

Exploratory correlation analysis (Figure 4) showed there were a number of significant monotonic associations (absolute values of Spearman rank correlation coefficients varied from 0.22 to 0.38) between activity metrics and continuous biomarker values. Only 6 of 10 cardiometabolic biomarkers were significantly associated with activity tracker–based metrics: BMI, waist circumference, waist-to-hip ratio, SBP, HDL, and TG. Almost all activity metrics were associated with some biomarkers, except for moderate-intensity PA and cdHR. Sedentary time, NightHR, dRHR, and IS had the greatest number of significant associations with biomarkers.

Further regression analysis showed that a number of activity metrics were significantly associated with BMI, waist circumference, waist-to-hip ratio, HDL, and TG, although there were no significant associations for LDL, BG, blood pressure, and total cholesterol (Table 3).

Blood biomarkers of cardiometabolic disease were significantly associated with steps-based metrics only. The number of daily steps was negatively associated with TG (beta=−6.8 per 1000 steps; 95% CI −13.0 to −0.6; *P*=.04), so participants with more daily steps had lower levels of TG. IS was negatively associated with TG (beta=−27.7 per 10% change; 95% CI −48.4 to −7.0; *P*=.01) and positively associated with HDL (beta=5.4 per 10% change; 95% CI 1.8 to 9.0; *P*=.005), so participants with higher IS had higher levels of HDL and lower levels of TG. Note that the significant associations between IS and blood biomarkers were independent of all confounders, including shift work. Patterns of circadian activity were also significantly associated with blood biomarkers of cardiometabolic disease independent of all confounders, including shift work. Participants from cluster *B*, whose daily activity was distributed more evenly without clear peaks, had worse indicators of cardiometabolic health, lower HDL (beta=−9.7; 95% CI −17.4 to −2.0; *P*=.02) and higher TG (beta=66.0; 95% CI 24.1 to 107.9; *P*=.003), compared with participants from clusters *A* and *C* with clear 2- or 3-peak patterns. Subsequent pairwise multiple analysis for HDL showed that IS remained a significant predictor when additionally adjusted for cluster membership, whereas cluster membership became nonsignificant (see Multimedia Appendix 5). Pairwise multiple regression analysis for TG indicated that IS remained a significant predictor after adjustment for steps and cluster membership; steps remained significant after adjustment for cluster membership, but it lost significance after adjusting for IS; and cluster membership remained a significant predictor after adjusting for IS and steps (see Multimedia Appendix 5).

Body composition biomarkers of cardiometabolic disease were significantly associated with metrics based on energy expenditure and HR-based metrics. Sedentary time was positively associated with BMI (beta=.1; 95% CI 0.003 to 0.2; *P*=.047), so participants with more sedentary time had a higher BMI. However, this association had borderline significance. Vigorous-intensity PA was positively associated with BMI (beta=.7; 95% CI 0.2 to 1.1; *P*=.01) and waist circumference (beta=1.9; 95% CI 0.6 to 3.2; *P*=.005), so participants with more time spent in vigorous-intensity PA had a higher BMI and a higher waist circumference. RHR was positively associated only with waist-to-hip ratio (beta=.02; 95% CI 0.001 to 0.03; *P*=.04), whereas dRHR (the difference between overall average HR and RHR) was negatively associated with BMI (beta=−0.5; 95% CI −1.0 to −0.1; *P*=.01) and waist circumference (beta=−1.3; 95% CI −2.4 to −0.2; *P*=.03). Lower RHR in relation to overall HR was associated with lower BMI and waist circumference values. Subsequent pairwise multiple analysis for BMI indicated that sedentary time together with vigorous-intensity PA remained significant predictors, as well as vigorous-intensity PA together with dRHR, whereas sedentary time paired with dRHR became nonsignificant (see Multimedia Appendix 5). Pairwise multiple analysis for waist circumference demonstrated that vigorous-intensity PA together with dRHR remained significant predictors after mutual adjustment (see Multimedia Appendix 5).

**Table 3:** Associations between wearable-based metrics of physical activity and common cardiometabolic disease risk markers.

Activity tracker-based metrics^a^	Blood biomarkers				Body composition					
	High-density lipoprotein		Triglyceride		BMI		Waist circumference		Waist-to-hip ratio	
	Beta (95% CI)	*P* value	Beta (95% CI)	*P* value	Beta (95% CI)	*P* value	Beta (95% CI)	*P* value	Beta (95% CI)	*P* value
Steps (×1000)	—^b^	—	−6.8 (−13.0 to −0.6)	.04	—	—	—	—	—	—
Interdaily stability of locomotor activity rhythm (×0.1)	5.4 (1.8 to 9.0)	.005	−27.7 (−48.4 to −7.0)	.01	—	—	—	—	—	—
Cluster B	−9.7 (−17.4 to −2.0)	.02	66.0 (24.1 to 107.9)	.003	—	—	—	—	—	—
Sedentary time, minutes (×10)	—	—	—	—	0.1 (0.003 to 0.2)	.047	—	—	—	—
Vigorous-intensity physical activity, minutes (×10)	—	—	—	—	0.7 (0.2 to 1.1)	.01	1.9 (0.6 to 3.2)	.005	—	—
Resting heart rate, bpm (×10)	—	—	—	—	—	—	—	—	0.02 (0.001 to 0.03)	.04
Delta of resting heart rate, bpm	—	—	—	—	−0.5 (−1.0 to −0.1)	.01	−1.3 (−2.4 to −0.2)	.03	—	—

^a^The table shows unstandardized coefficients (beta), 95% CI, and exact *P* values of activity metrics as predictors of cardiometabolic disease biomarkers in multiple linear regression models. For each predictor, adjustments were made for age, gender, ethnicity, education level, and shift work. Only significant coefficients reported. Coefficients of adjusted covariates are omitted. The full results are provided in Multimedia Appendix 4. For steps, the effect is for each additional 1000 steps; for IS, the effect is for each 0.1 change in score; for sedentary time and vigorous-intensity physical activity, the effects are for each additional 10 min of time spent in respective activity; for RHR, the effect is for each additional 10 bpm.

^b^Nonsignificant coefficients are omitted. The full results are provided in Multimedia Appendix 4.

Light-intensity PA, moderate-intensity PA, MVPA, average HR, DayHR, NightHR, cdHR, and IV did not indicate any significant associations with cardiometabolic disease biomarkers.

## Discussion

### Principal Findings

We explored the relationships between metrics of locomotor activity and regularity in circadian rhythms derived from a consumer-grade fitness tracker and risk biomarkers of cardiometabolic disease in a multiethnic Asian working population in Singapore. We found that blood biomarkers of cardiometabolic disease (HDL cholesterol and TGs) were significantly associated with steps-based activity metrics (daily steps, IS, and different patterns of circadian activity), whereas body composition biomarkers (BMI, waist circumference, and waist-to-hip ratio) were significantly associated with energy expenditure–based and HR-based metrics (sedentary time, vigorous-intensity PA, RHR, and dRHR). These associations were significant, independent of age, gender, ethnicity, education, and shift work. One of our principal findings is that the stability of circadian activity rhythm was significantly associated with blood biomarkers of cardiometabolic health independent of shift work, which along with jet lag is considered the strongest disruptor of normal circadian biorhythms and bears a higher risk of developing cardiovascular diseases [55,56], metabolic syndrome, and type 2 diabetes [57,58]. Moreover, the association between IS of circadian rhythm and HDL cholesterol and TGs did not depend on the overall activity level (the number of daily steps), which indicates that a stable activity rhythm may be beneficial for cardiometabolic health at any level of activity. Patterns of circadian activity were also strongly associated with blood biomarkers, which means that some daily regimes may be more beneficial for cardiometabolic health than others regardless of the stability of these regimes. Finally, we found that vigorous-intensity PA was positively associated with BMI and waist circumference, meaning that participants who spent more time engaging in vigorous-intensity PA had higher BMI and waist circumference. Overall, these results contribute to the sparse but growing evidence on, first, the benefits of regular and stable locomotor activity for cardiometabolic health, and, second, the potential public health value of consumer-grade fitness trackers in population health monitoring and cardiometabolic disease risk prediction.

### Strengths and Limitations

This study has several strengths. First, a reasonably long period of continuous activity tracking (21 days) was involved, which enabled a more precise assessment of the stability and regularity of circadian activity as well as the level of habitual PA and HR in different states and conditions. Second, we used a wide range of measures based on different wearable data, which evaluated everyday activity in a comprehensive way. Third, we used IS, a nonparametric measure of the activity rhythm, instead of more common parametric measures. IS is not based on any assumptions about the pattern of the rhythm and hence allows assessment and comparison of the regularity of circadian activity between individuals with different lifestyles. Fourth, regarding clinical measurements, we followed standard operating procedures and collected fasting blood samples, which allowed us to use TGs and fasting BG as biomarkers. Finally, in the statistical analysis, we used linear regression models adjusted for a range of potential confounders, including age, gender, ethnicity, education, and shift work, which allowed us to robustly estimate the linear effect of wearable-derived predictors on continuous measures of biomarkers.

The main limitations of the study are the relatively small sample size (N=86), time gap (3 months on average) between clinical measurements and activity tracking period, and the limited accuracy of Fitbit wearables in measuring energy-expenditure metrics. As we studied a working population that did not change their job over the course of the study, it is acceptable to assume that their behavior and activity patterns were habitual and stable over time. Although we cannot exclude the influence of other multiple contextual factors on the locomotor activity of participants between clinical measurements and activity tracking, such a time gap is not unusual in cohort studies that include an activity tracking component [26]. We found that study participants were highly active in terms of time spent in MVPA, having on average 237 min per day of MVPA and more than 30 min per day of vigorous-intensity PA, much more than meeting PA guidelines [54]. These estimates should be interpreted with caution because Fitbit wearables tend to progressively overestimate the time spent in higher intensity activities [49]. Another limitation is the threshold of a minimum daily wearing time of Fitbit trackers to include a day into the analysis—set to at least 18 hours per day (75% of daily time). This threshold may affect the results of the analysis because missing 6 hours per day is still a significant gap. However, the use of the less strict time gap compared with, for example, 4 hours that is common in actigraphy research brings our study closer to real-life conditions and enables us to explore the potential of consumer-grade wearables in health risk prediction more realistically. In addition, given that the overall observed level of activity (steps) is quite high, we assume that most missing hours did not affect the active phases of the monitored days. An additional limitation concerns the definitions of HR-based metrics, which should be appraised with caution because there is no formal theory-based rationale underpinning them. For example, sampling HR between 2 pm and 4 pm for measuring DayHR can be affected by proximity to a postprandial period or other daily events. Finally, this study has a cross-sectional design, which limits the possibility of making causal inferences.

### Comparison With Previous Research

The results of our study are mostly consistent with previous research. First of all, data of fitness tracker–based metrics obtained in our study, namely daily steps and RHR, were similar to the data from the recent study in the same population (Singapore residents) [41].

Second, our findings complement the extant literature on the effects of objectively measured sedentary time and PA on cardiovascular and metabolic health. We found that more daily steps were significantly associated with lower TG after adjustment for confounders, which is consistent with a previous study [41]. Furthermore, correlation analysis indicated that longer daily sedentary time was associated with higher BMI, higher waist circumference, higher waist-to-hip ratio, higher TG levels, and lower HDL [11-22]. However, contrary to previous research, only the association between sedentary time and BMI remained marginally significant after adjustments for age, gender, ethnicity, education, and shift work in the regression models. We hypothesize that this may be because of insufficient power of our study compared with previous research as well as because of the lower accuracy in measuring calorie expenditure by Fitbit wearables compared with research-grade actigraphs. In addition, we did not find associations between cardiometabolic disease biomarkers and light-intensity PA, moderate-intensity PA, or MVPA. Moreover, contrary to previous findings, our analysis showed that participants spending more time engaging in vigorous-intensity PA had a higher BMI and waist circumference. This may potentially be explained by overweight participants already being concerned with their body condition and thus spending more time exercising during the study. Alternatively, this finding may potentially be related to the limited accuracy of Fitbit wearables in measuring energy expenditure and the tendency to overestimate the time spent in higher intensity activities [49], which may have substantial impact on the result.

Third, we found that RHR metrics were associated with body composition markers of cardiometabolic disease risk, including BMI, waist circumference, and waist-to-hip ratio, but not with blood biomarkers, which partially replicates earlier evidence [3,41]. Note that RHR was associated only with waist-to-hip ratio, whereas dRHR was associated with BMI and waist circumference. A lower number of significant associations might be explained by the insufficient power of the sample size (86 vs 233 participants) or the time gap between clinical measurements and activity tracking. However, having a longer period of activity tracking compared with the study by Lim et al [41] (21 days over 3 days) suggests that our estimates of RHR are more stable than their study. RHR sampled and estimated within a 3-day period is more sensitive to random fluctuations, for example, because of higher activity on 1 day, and, therefore, can have more skewed distribution compared with RHR obtained from a longer period.

Finally, harnessing longer activity tracking, we explored the effects of regularity in circadian activity rhythms on cardiometabolic disease biomarkers, going beyond standard metrics available from consumer-grade fitness trackers. Nevertheless, our findings are consistent with the previous studies investigating the clinical value of activity rhythms in respect of cardiometabolic risk or related biomarkers [23,24]. Our data indicate a strong positive association of IS with HDL cholesterol and a significant negative association with TG, both independent of sociodemographic confounding factors and when adjusted for steps or different activity patterns. Despite a much smaller sample size, our study has several advantages in contrast to previous research. First, both studies were limited by an elderly population, and one study considered only older men, whereas we studied working-age population (aged 21-65 years). Second, both studies used research-grade actigraphs, whereas we used consumer-grade fitness trackers and obtained similar results. Consumer-grade wearables are much more affordable and common among the general population and, therefore, have a higher potential value for public health, enabling predictive health monitoring on a population scale. Third, the duration of activity tracking in both studies did not exceed 7 days, whereas we collected wearables’ data for 21 days. Longer tracking enables one to more precisely infer an average profile of daily activity and estimate the IS. Fourth, only Sohail et al [24] directly analyzed relationships between IS and cardiometabolic disease risk markers, but they collected nonfasting blood samples and so could not investigate associations with TG and BG. Thus, our study is the first to report a significant association between steps-based IS and TG, an important blood biomarker of metabolic syndrome.

### Possible Mechanisms

How might rhythms of locomotor activity be related to cardiometabolic health? Circadian rhythms are cyclic biochemical processes with a 24-hour period, which play a crucial role in physiology and metabolism [59]. Circadian biorhythms are controlled by molecular circadian clocks, which are present in many cells and synchronize internal biological functions with environmental conditions [60]. External stimuli, called Zeitgebers, mainly light and food intake, can change the phase of a circadian oscillator and entrain circadian clocks. Hence, the misalignment of Zeitgebers and intrinsic circadian clocks entail consequences for metabolic processes and health. Experimental studies in humans demonstrated that circadian misalignment (shift of active phase from habitual time) imposes adverse cardiometabolic implications—decreased leptin, increased glucose, increased arterial pressure [61], and also disruption in free fatty acids and TGs [62]. Disruption of normal circadian rhythms because of shift work, chronic jetlag, artificial light, or poor sleep impacts human health and increases the risk of many diseases, including obesity, type 2 diabetes, and various cardiovascular diseases [56,63,64]. According to a recent review, locomotor activity modulates the molecular clock in skeletal muscle, affecting both the amplitude and phase of circadian rhythms [65]. Besides, it was found that the muscle molecular clock does not synchronize rapidly with the changes in the behavioral cycle [62]. Here, relying on previous research and our findings, we cautiously assume that unstable locomotor activity rhythms, even because of commuting to work, breaks, or any other locomotor activity occurring at different times, do not allow circadian clocks to align with these activity rhythms and establish a stable internal biorhythm and, therefore, provoke disruptions in metabolic functioning. Moreover, as IS is a nonparametric measure, we may assume that stable and regular rhythms in locomotor activity, regardless of shift work and alignment with day-night cycle, contribute to better cardiometabolic outcomes.

### Conclusions and Future Research

Wearable fitness trackers enable the collection of biometric data, such as steps, HR, and sleep characteristics, at a low cost and at a population scale, which might have clinical value and public health implications. Our findings suggest that consumer-grade fitness trackers can provide insightful information with respect to the risk factors of cardiometabolic disease. We employed the measure of IS in circadian activity rhythms based on steps and show that this metric can be used for personalized risk prediction. With wearables, people can monitor their biometrics and activity, enabling early detection of deviations in digital biomarkers. In addition, wearables can be used to increase control over modifiable behavioral and lifestyle risk factors of cardiometabolic disease.

The molecular mechanisms underlying the effects of activity rhythms on the risk biomarkers of cardiometabolic disease require additional longitudinal and experimental studies, and results need to be confirmed in other populations. Future research examining the utility of consumer-grade fitness trackers should focus on the prognostic prediction of health outcomes in free-living conditions using wearable data and recent advances in machine learning. The development of highly accurate predictive algorithms that combine different data and digital markers in a single model is one of the main targets in this field.

## Appendix

Multimedia Appendix 1 R code for Fitbit data processing and analysis.

Multimedia Appendix 2 Differences in activity tracker–based metrics between shift and nonshift workers.

Multimedia Appendix 3 Analysis of stability of resting heart rate sampled from 3-day windows (*P* values of Kolmogorov-Smirnov tests).

Multimedia Appendix 4 Full results of regression analysis.

Multimedia Appendix 5 Pairwise multiple regression analysis.

## Abbreviations

BG: blood glucose
cdHR: circadian delta of heart rate
CRP: C-reactive protein
DayHR: daytime heart rate
DBP: diastolic blood pressure
dRHR: delta of resting heart rate
HDL: high-density lipoprotein
HR: heart rate
IS: interdaily stability
IV: interdaily variation
LDL: low-density lipoprotein
MET: metabolic equivalent
MVPA: moderate-to-vigorous physical activity
NightHR: nighttime heart rate
NTU: Nanyang Technological University
PA: physical activity
RHR: resting heart rate
SBP: systolic blood pressure
TG: triglyceride
