# Supplementary material for: Activity Tracker–Based Metrics as Digital Markers of Cardiometabolic Health in Working Adults: Cross-Sectional Study
Source: JMIR Mhealth Uhealth. 2020 Jan 31;8(1):e16409. doi: 10.2196/16409 (PMC7055791; doi:10.2196/16409)
Supplement: Multimedia Appendix 2 [file mhealth_v8i1e16409_app2.pdf]

Multimedia Appendix 2. Differences in activity-tracker-based metrics between shift and non-shift workers.

Activity tracker-based metrics	Non-shift workers N=42		Shift workers N=41		
	Mean	SD	Mean	SD	*P*-value
Steps	11260.0	2934.4	10459.9	2574.5	.16 [a]
Sedentary time	754.9	104.2	821.7	82.3	<.001 [a]
Light PA	1205.7	59.4	1200.1	61.7	.91 [a]
Moderate PA	203.6	54.9	207.3	56.0	.99 [a]
Vigorous PA	30.7	14.3	32.7	17.8	.77 [a]
MVPA	234.3	59.4	239.9	61.7	.91 [a]
IS	0.34	0.10	0.23	0.08	<.001 [a]
IV	1.30	0.23	13.4	0.22	.58 [a]
HR	75.5	6.8	77.1	8.3	.60 [a]
RHR	69.6	7.4	71.6	8.8	.48 [a]
dRHR	5.9	2.1	5.5	1.8	.20 [a]
Day HR	80.4	7.8	82.5	10.3	.52 [a]
Night HR	62.0	7.1	66.2	8.6	.01 [a]
cdHR	18.3	4.9	16.4	6.3	.19 [a]
	N		N		
Cluster A	28		7		<.001 [b]
Cluster B	7		27		
Cluster C	7		7		

PA: Physical Activity; MVPA: moderate-to-vigorous physical activity; IS: interdaily stability of locomotor activity rhythm; IV: interdaily variation of locomotor activity; HR: heart rate; RHR: resting heart rate; dRHR: delta of resting heart rate; Day HR: daytime heart rate; Night HR: nighttime heart rate; cdHR: circadian delta of heart rate.

[a] P-values obtained from Kruskal-Wallis rank-sum test for non-normally distributed continuous variable.

[b] P-value obtained from Pearson's Chi-squared test.
