# Supplementary material for: Activity Tracker–Based Metrics as Digital Markers of Cardiometabolic Health in Working Adults: Cross-Sectional Study
Source: JMIR Mhealth Uhealth. 2020 Jan 31;8(1):e16409. doi: 10.2196/16409 (PMC7055791; doi:10.2196/16409)
Supplement: Multimedia Appendix 4 [file mhealth_v8i1e16409_app4.pdf]

Multimedia Appendix 4. Full results of multiple regression analysis.

	Health outcomes									
	BMI	Waist circumference	Wasit-hip ratio	HDL	LDL	TG	BG	SBP	DBP	Total cholesterol
	N=83	N=82	N=82	N=70	N=69	N=69	N=69	N=82	N=82	N=70
	β (95% CI)	β (95% CI)	β (95% CI)	β (95% CI)	β (95% CI)	β (95% CI)	β (95% CI)	β (95% CI)	β (95% CI)	β (95% CI)
Steps	-0.0003 (-0.001, 0.00002)	-0.001 (-0.001, 0.00005)	-0.00000 (-0.00001, 0.00000)	0.001 (-0.0001, 0.002)	-0.001 (-0.004, 0.001)	-0.007 (-0.013, -0.001)	-0.001 (-0.002, 0.0002)	0.0001 (-0.001, 0.001)	-0.0002 (-0.001, 0.001)	-0.002 (-0.005, 0.001)
	P=.07	*P*=.07	*P*=.20	*P*=.09	*P*=.33	*P*=.04[a]	*P*=.10	*P*=.91	*P*=.58	*P*=.12
Sedentary time	0.010 (0.0003, 0.019)	0.024 (-0.001, 0.048)	0.0001 (-0.0001, 0.0002)	-0.030 (-0.065, 0.005)	0.053 (-0.021, 0.128)	0.123 (-0.078, 0.323)	0.016 (-0.023, 0.055)	0.019 (-0.014, 0.051)	0.022 (-0.003, 0.048)	0.069 (-0.022, 0.160)
	P=.047[a]	*P*=.06	*P*=.36	*P*=.10	*P*=.17	*P*=.24	*P*=.42	*P*=.26	*P*=.09	*P*=.14
Light PA	0.001 (-0.013, 0.015)	-0.004 (-0.041, 0.033)	-0.0001 (-0.0003, 0.0001)	-0.002 (-0.055, 0.051)	-0.008 (-0.119, 0.103)	-0.003 (-0.300, 0.294)	-0.016 (-0.073, 0.042)	-0.010 (-0.059, 0.038)	-0.002 (-0.041, 0.037)	0.007 (-0.130, 0.143)
	P=.84	*P*=.83	*P*=.19	*P*=.94	*P*=.89	*P*=.98	*P*=.60	*P*=.68	*P*=.92	*P*=.92
Moderate PA	-0.008 (-0.023, 0.008)	-0.012 (-0.053, 0.028)	0.0001 (-0.0001, 0.0003)	0.002 (-0.056, 0.059)	-0.0004 (-0.121, 0.120)	0.076 (-0.248, 0.400)	0.014 (-0.049, 0.077)	-0.003 (-0.056, 0.050)	-0.005 (-0.047, 0.038)	-0.001 (-0.150, 0.148)
	P=.33	*P*=.56	*P*=.37	*P*=.96	*P*=.99	*P*=.65	*P*=.66	*P*=.91	*P*=.84	*P*=.99
Vigorous PA	0.066 (0.017, 0.115)	0.194 (0.064, 0.324)	0.001 (-0.00000, 0.001)	0.009 (-0.193, 0.211)	0.122 (-0.299, 0.542)	-0.887 (-1.998, 0.225)	0.053 (-0.167, 0.273)	0.177 (0.001, 0.352)	0.079 (-0.063, 0.221)	-0.085 (-0.606, 0.435)
	P=.011[a]	*P*=.005 [b]	*P*=.06	*P*=.93	*P*=.57	*P*=.12	*P*=.64	*P*=.05	*P*=.28	*P*=.75
MVPA	-0.001 (-0.015, 0.013)	0.004 (-0.033, 0.041)	0.0001 (-0.0001, 0.0003)	0.002 (-0.051, 0.055)	0.008 (-0.103, 0.119)	0.003 (-0.294, 0.300)	0.016 (-0.042, 0.073)	0.010 (-0.038, 0.059)	0.002 (-0.037, 0.041)	-0.007 (-0.143, 0.130)
	P=.84	*P*=.83	*P*=.19	*P*=.94	*P*=.89	*P*=.98	*P*=.60	*P*=.68	*P*=.92	*P*=.92
IS	-7.636 (-17.810, 2.538)	-23.783 (-50.898, 3.332)	-0.146 (-0.292, -0.001)	54.343 (18.210, 90.475)	17.103 (-64.038, 98.244)	-276.768 (-483.716, -69.820)	-40.327 (-81.486, 0.832)	-18.487 (-54.457, 17.484)	-14.654 (-43.286, 13.979)	-10.850 (-110.235, 88.535)
	P=.15	*P*=.09	*P*=.05	*P*=.005 [b]	*P*=.68	*P*=.012[a]	*P*=.06	*P*=.32	*P*=.32	*P*=.83

IV	0.492 (-4.034, 5.019)	-2.002 (-13.948, 9.944)	-0.020 (-0.085, 0.044)	-2.844 (-21.302, 15.613)	14.467 (-23.948, 52.882)	10.413 (-93.218, 114.043)	0.615 (-19.513, 20.742)	-7.858 (-23.412, 7.697)	-2.056 (-14.510, 10.398)	18.725 (-28.645, 66.094)
	P=.83	*P*=.74	*P*=.55	*P*=.76	*P*=.46	*P*=.85	*P*=.95	*P*=.33	*P*=.75	*P*=.44
Cluster B	0.781 (-1.306, 2.868)	1.768 (-3.771, 7.307)	0.013 (-0.017, 0.043)	-9.712 (-17.386, -2.038)	10.400 (-6.213, 27.014)	65.973 (24.104, 107.841)	2.451 (-6.229, 11.131)	-3.858 (-11.079, 3.363)	-3.045 (-8.784, 2.693)	10.792 (-9.717, 31.301)
	P=.47	*P*=.53	*P*=.39	*P*=.016[a]	*P*=.23	*P*=.003 [b]	*P*=.58	*P*=.30	*P*=.30	*P*=.31
Cluster C	0.750 (-1.646, 3.145)	0.368 (-5.968, 6.705)	-0.004 (-0.038, 0.030)	-4.142 (-13.262, 4.977)	9.025 (-10.691, 28.740)	50.090 (0.403, 99.776)	-3.951 (-14.252, 6.349)	-2.144 (-10.406, 6.117)	-2.998 (-9.563, 3.568)	12.458 (-11.915, 36.830)
	P=.54	*P*=.91	*P*=.82	*P*=.38	*P*=.37	*P*=.05	*P*=.46	*P*=.61	*P*=.37	*P*=.32
HR	0.030 (-0.079, 0.139)	0.120 (-0.169, 0.410)	0.001 (-0.0001, 0.003)	-0.145 (-0.582, 0.292)	-0.171 (-1.139, 0.796)	1.844 (-0.716, 4.404)	0.306 (-0.193, 0.806)	0.274 (-0.102, 0.650)	0.215 (-0.084, 0.515)	0.783 (-0.330, 1.896)
	P=.59	*P*=.42	*P*=.06	*P*=.52	*P*=.73	*P*=.16	*P*=.23	*P*=.16	*P*=.16	*P*=.17
RHR	0.057 (-0.045, 0.159)	0.180 (-0.092, 0.451)	0.002 (0.0001, 0.003)	-0.229 (-0.640, 0.183)	-0.137 (-1.056, 0.782)	2.251 (-0.153, 4.656)	0.360 (-0.111, 0.831)	0.290 (-0.063, 0.643)	0.235 (-0.046, 0.516)	0.780 (-0.271, 1.832)
	P=.28	*P*=.20	*P*=.04[a]	*P*=.28	*P*=.77	*P*=.07	*P*=.14	*P*=.11	*P*=.11	*P*=.15
dRHR	-0.538 (-0.952, -0.124)	-1.275 (-2.386, -0.165)	-0.004 (-0.010, 0.002)	1.612 (-0.014, 3.238)	-0.254 (-3.768, 3.261)	-8.609 (-17.801, 0.583)	-1.223 (-3.031, 0.585)	-0.808 (-2.300, 0.684)	-0.754 (-1.939, 0.430)	-1.279 (-5.584, 3.026)
	P=.013[a]	*P*=.028[a]	*P*=.22	*P*=.06	*P*=.89	*P*=.07	*P*=.19	*P*=.29	*P*=.22	*P*=.56
Day HR	0.001 (-0.089, 0.092)	0.026 (-0.213, 0.266)	0.001 (-0.0004, 0.002)	-0.137 (-0.482, 0.208)	-0.078 (-0.830, 0.673)	1.534 (-0.449, 3.518)	0.310 (-0.075, 0.694)	0.106 (-0.207, 0.419)	0.125 (-0.123, 0.373)	0.576 (-0.304, 1.457)
	P=.96	*P*=.83	*P*=.20	*P*=.44	*P*=.84	*P*=.14	*P*=.12	*P*=.51	*P*=.33	*P*=.21
Night HR	0.050 (-0.059, 0.159)	0.183 (-0.103, 0.470)	0.002 (0.00001, 0.003)	-0.369 (-0.808, 0.070)	-0.022 (-1.001, 0.958)	2.457 (-0.101, 5.015)	0.272 (-0.235, 0.778)	0.327 (-0.045, 0.698)	0.188 (-0.110, 0.487)	0.750 (-0.391, 1.891)
	P=.37	*P*=.22	*P*=.05	*P*=.11	*P*=.97	*P*=.06	*P*=.30	*P*=.09	*P*=.22	*P*=.20
cdHR	-0.088 (-0.236, 0.059)	-0.273 (-0.666, 0.120)	-0.001 (-0.003, 0.002)	0.246 (-0.348, 0.839)	-0.181 (-1.431, 1.068)	0.237 (-3.121, 3.595)	0.413 (-0.231, 1.057)	-0.325 (-0.840, 0.191)	-0.012 (-0.427, 0.403)	0.384 (-1.151, 1.920)
	P=.25	*P*=.18	*P*=.63	*P*=.42	*P*=.78	*P*=.89	*P*=.21	*P*=.22	*P*=.96	*P*=.63

[a]$P<0.05$; [b]$P<0.01$

The table shows unstandardized coefficients (β), 95% confidence intervals and exact P values of activity metrics as predictors of cardiometabolic disease biomarkers in multiple linear regression models. For each predictor adjustments were made for age, gender, ethnicity, education level and shift work. CI:

Confidence Interval; BMI: body mass index; HDL: high-density lipoprotein cholesterol; LDL: low-density lipoprotein cholesterol; TG: triglyceride; BG: blood glucose; SBP: systolic blood pressure; DBP: diastolic blood pressure; PA: physical activity; MVPA: moderate-to-vigorous physical activity; IS: interdaily stability of locomotor activity rhythm; IV: interdaily variation of locomotor activity; HR: heart rate; RHR: resting heart rate; dRHR: delta of resting heart rate; Day HR: daytime heart rate; Night HR: nighttime heart rate; cdHR: circadian delta of heart rate
