# Supplementary material for: Activity Tracker–Based Metrics as Digital Markers of Cardiometabolic Health in Working Adults: Cross-Sectional Study
Source: JMIR Mhealth Uhealth. 2020 Jan 31;8(1):e16409. doi: 10.2196/16409 (PMC7055791; doi:10.2196/16409)
Supplement: Multimedia Appendix 5 [file mhealth_v8i1e16409_app5.pdf]

Multimedia Appendix 5. Pairwise multiple regression analysis.

Table. 1. Multiple regression analysis for HDL.

	HDL<br>β (95% CI)
IS (1/10)	4.5 (0.8, 8.3)<br>*P* = .02
Cluster B	-7.2 (-14.9, 0.5)<br>*P* = .07
Cluster C	-4.0 (-12.8, 4.8)<br>*P* = .37
Observations	70
R^2	0.48
Adjusted R^2	0.40

The table shows unstandardized coefficients (β), confidence intervals and exact *P* values of activity metrics as predictors of HDL in multiple linear regression model. The model was adjusted for age, gender, ethnicity, education level and shift work. Estimates of adjusted covariates are omitted. For IS the effect is for each 0.1 change in score. Cluster A was a reference category. CI: confidence interval; HDL: high-density lipoprotein cholesterol; IS: interdaily stability of locomotor activity rhythm.

Table. 2. Multiple regression analysis for TG.

		TG	
	β (95% CI)	β (95% CI)	β (95% CI)
IS (1/10)	-23.4 (-44.5, -2.3)<br>*P* = .03	-21.5 (-42.3, -0.6)<br>*P* = .049	
Steps (x1000)	-5.0 (-11.0, 1.0)<br>*P* = .12		-8.0 (-14.0, -2.0)<br>*P* = .01
Cluster B		53.5 (10.9, 96.1)<br>*P* = .02	70.2 (30.2, 110.3)<br>*P* = .002
Cluster C		49.2 (0.7, 97.7)<br>*P* = .05	54.9 (7.3, 102.4)<br>*P* = .03
Observations	69	69	69
R^2	0.30	0.35	0.38
Adjusted R^2	0.21	0.25	0.28

The table shows unstandardized coefficients (β), confidence intervals and exact *P* values of activity metrics as predictors of TG in multiple linear regression models. Each model with a pair of activity metrics as predictors were adjusted for age, gender, ethnicity, education level and shift work. Estimates of adjusted covariates are omitted. For IS the effect is for each 0.1 change in score; for steps, the effect is for each additional 1,000 steps. Cluster A was a reference category. CI: confidence interval; TG: triglyceride.

Table. 3. Multiple regression analysis for BMI.

	BMI		
	β (95% CI)	β (95% CI)	β (95% CI)
Sedentary, mins (x10)	0.1 (0.03, 0.2)	0.03 (-0.1, 0.15)	
	P = .01	*P* = .60	
Vigorous PA, mins (x10)	0.8 (0.3, 1.3)		1.0 (0.6, 1.6)
	P = .003		*P* < .001
dRHR, bpm		-0.4 (-1.0, 0.1)	-0.9 (-1.3, -0.5)
		P = .12	*P* < .001
Observations	83	83	83
R^2	0.47	0.41	0.53
Adjusted R^2	0.41	0.35	0.48

The table shows unstandardized coefficients (β), confidence intervals and exact *P* values of activity metrics as predictors of BMI in multiple linear regression models. Each model with a pair of activity metrics as predictors were adjusted for age, gender, ethnicity, education level and shift work. Estimates of adjusted covariates are omitted. For sedentary time and vigorous PA, the effects are for each additional 10 minutes of time spent in respective activity. CI: confidence interval; BMI: body mass index; dRHR: delta of resting heart rate; PA: physical activity; bpm: beats per minute.

Table. 4. Multiple regression analysis for waist circumference.

	Waist circumference
	β (95% CI)
Vigorous PA, mins (x10)	2.9 (1.6, 4.2)
	P < .001
dRHR, bpm	-2.2 (-3.2, -1.1)
	P < .001
Observations	82
R^2	0.64
Adjusted R^2	0.60

The table shows unstandardized coefficients (β), confidence intervals and exact *P* values of activity metrics as predictors of waist circumference in multiple linear regression model. The model was adjusted for age, gender, ethnicity, education level and shift work. Estimates of adjusted covariates are omitted. For vigorous PA, the effect is for each additional 10 minutes. CI: confidence interval; dRHR: delta of resting heart rate; PA: physical activity; bpm: beats per minute.
